# National data system on near miss and maternal death: shifting from maternal risk to public health impact in Nigeria

**DOI:** 10.1186/1742-4755-6-8

**Published:** 2009-06-09

**Authors:** Olufemi T Oladapo, Olalekan O Adetoro, Oluwarotimi Fakeye, Bissallah A Ekele, Adeniran O Fawole, Aniekan Abasiattai, Oluwafemi Kuti, Jamilu Tukur, Adedapo BA Ande, Olukayode A Dada

**Affiliations:** 1 Maternal and Fetal Health Research Unit, Department of Obstetrics & Gynaecology, Olabisi Onabanjo University Teaching Hospital, Sagamu, Ogun State, Nigeria; 2 Centre for Research in Reproductive Health, Sagamu, Ogun State, Nigeria; 3 Department of Obstetrics & Gynaecology, University of Ilorin Teaching Hospital, Ilorin, Kwara State, Nigeria; 4 Department of Obstetrics & Gynaecology, Usman Danfodiyo University Teaching Hospital, Sokoto, Sokoto State, Nigeria; 5 Department of Obstetrics & Gynaecology, University College Hospital, Ibadan, Oyo State, Nigeria; 6 Department of Obstetrics & Gynaecology, University of Uyo Teaching Hospital, Uyo, Akwa-Ibom State, Nigeria; 7 Department of Obstetrics, Gynaecology & Perinatology, Obafemi Awolowo University Teaching Hospital Complex, Ile-Ife, Osun State, Nigeria; 8 Department of Obstetrics & Gynaecology, Aminu Kano Teaching Hospital, Kano, Kano State, Nigeria; 9 Department of Obstetrics & Gynaecology, University of Benin Teaching Hospital, Benin-City, Edo State, Nigeria

## Abstract

**Background:**

The lack of reliable and up-to-date statistics on maternal deaths and disabilities remains a major challenge to the implementation of Nigeria's *Road Map to Accelerate the Millennium Development Goal related to Maternal Health (MDG-5)*. There are currently no functioning national data sources on maternal deaths and disabilities that could serve as reference points for programme managers, health advocates and policy makers. While awaiting the success of efforts targeted at overcoming the barriers facing establishment of population-based data systems, referral institutions in Nigeria can contribute their quota in the quest towards MDG-5 by providing good quality and reliable information on maternal deaths and disabilities on a continuous basis. This project represents the first opportunity to initiate a scientifically sound and reliable quantitative system of data gathering on maternal health profile in Nigeria.

**Objective:**

The primary objective is to create a national data system on maternal near miss (MNM) and maternal mortality in Nigerian public tertiary institutions. This system will conduct periodically, both regionally and at country level, a review of the magnitude of MNM and maternal deaths, nature of events responsible for MNM and maternal deaths, indices for the quality of care for direct obstetric complications and the health service events surrounding these complications, in an attempt to collectively define and monitor the standard of comprehensive emergency obstetric care in the country.

**Methods:**

This will be a nationwide cohort study of all women who experience MNM and those who die from pregnancy, childbirth and puerperal complications using uniform criteria among women admitted in tertiary healthcare facilities in the six geopolitical zones in Nigeria. This will be accomplished by establishing a network of all public tertiary obstetric referral institutions that will prospectively collect specific information on potentially fatal maternal complications. For every woman enrolled, the health service events (care pathways) within the facility will be evaluated to identify areas of substandard care/avoidable factors through clinical audit by the local research team. A summary estimate of the frequencies of MNM and maternal deaths will be determined at intervals and indicators of quality of care (case fatality rate, both total and cause-specific and mortality index) will be evaluated at facility, regional and country levels.

**Management:**

Overall project management will be from the Centre for Research in Reproductive Health (CRRH), Sagamu, Nigeria. There will be at least two meetings and site visits for efficient coordination of the project by regional coordinators and central coordinating staff. Data will be transferred electronically by hospital and regional coordinators and managed at the Data Management Unit of CRRH, Sagamu, Nigeria.

**Expected outcomes:**

The outcome of the study would provide useful information to the health practitioners, policy-makers and international partners on the strengths and weaknesses of the infrastructures provided for comprehensive emergency obstetric care in Nigeria. The successful implementation of this project will pave way for the long-awaited Confidential Enquiries into Maternal Deaths that would guide the formulation and or revision of obstetric policies and practices in Nigeria. Lessons learnt from the establishment of this data system can also be used to set up similar structures at lower levels of healthcare delivery in Nigeria.

## Background

In spite of the efforts of many international and developmental health agencies, maternal deaths and disabilities remain major public health problems in the developing countries. The little progress made towards the three-quarter reduction of the 1990 estimate of global maternal mortality ratio by 2015 can essentially be attributed to the meager achievements of low and middle income countries in this regard. Available data on maternal deaths from sub-Saharan Africa midway to the deadline of millennium declaration cast doubt on the realisation of its fifth goal [1,2].

While acknowledging the contributions of poor governmental commitment and inappropriate resource allocation to maternal health priorities, one major challenge to the realisation of the MDG-5 by developing countries is the lack of reliable data on maternal deaths and disabilities on which to measure progress made towards its attainment. This lack of reliable information limits the advocacy power of health practitioners and planners in sourcing for political commitment for appropriate distribution of resources that is necessary to tackle the problems underlying maternal ill health. For instance, in Nigeria, few population-based data on maternal deaths and disabilities exist, vital registrations of deaths are grossly unreliable as legal enforcement by government and socio-cultural values of the population are unfavourable for data gathering. Unlike what obtains in developed countries, there are presently no functioning national data sources on maternal deaths and disabilities in Nigeria which could serve as reference points for health advocates and policy makers. Estimates derived from statistical models by WHO and sister agencies have wide margins of uncertainty and are not reliable enough to assess the quality of maternal health care, monitor trend on the short term, determine health system priorities or base allocation of health resources. This problem is partly attributable to the technical and cost limitations of the methods for population-based measurement of maternal mortality and morbidity that cannot be readily overcome by a low resource country like Nigeria. Nevertheless, as MDG-5 has renewed attention to the issues of sources, quality and method of gathering data, it becomes important to devise mechanisms by which efforts to reduce maternal deaths and disabilities in Nigeria can be monitored.

An important measure of maternal mortality that is often overlooked but which is equally very important especially in areas with high incidence of pregnancy and childbirth related deaths is the absolute number of maternal deaths which emphasizes the *magnitude *rather than the *risk *of maternal death within a given population. The number of maternal deaths is a self-evident measure that gives a clear indication of the public health impact of maternal mortality [3]. Although, the measure is not always useful for between-country comparisons, in some circumstances, raw number can better express the situation. For instance, maternal deaths recorded over a period of three years in a single health facility serving a population of less than two million people in Nigeria [4] was close to half of the total number of maternal deaths recorded in the Confidential Enquiries into Maternal Deaths during the same period in the United Kingdom [5]. Similarly, Bangladesh recorded a total of 21,600 maternal deaths in a year compared to 500 in the United States whose population is two and half times that of Bangladesh's [6].

Besides maternal deaths, another quality indicator of obstetric care that has gained international attention in recent time is maternal near miss (MNM), otherwise referred to as severe acute maternal morbidity. According to the WHO, a maternal near miss case is a woman who nearly died but survived a complication that occurred during pregnancy, childbirth or within 42 days of termination of pregnancy [7]. MNM cases occur more frequently than maternal death and may generate more information as the woman herself can be a source of data. Although previously started in the developed countries where maternal mortality is low, it has also been found to provide useful information even in places with high maternal death rate [8]. Unlike in developed countries, maternal deaths in developing countries tend to result from regular life threatening complications and thus inclusion of MNM review in maternal death audit tend to provide a clearer picture and more robust conclusions on the pathway to maternal death [9,10]. Therefore, the study of data collected on MNM and maternal deaths has been found to be useful for the identification of health systems failures and a relevant source of information for policy makers in the selection of maternal health care priorities [8-10].

Although there is an assumption that maternal deaths recorded in the hospitals represent a fraction of the total maternal deaths as most women deliver outside health facilities, available data from many Nigerian centres indicate that large number of maternal deaths and MNM still occur within hospitals [11-13]. In view of the public health implications of the magnitude of MNM and maternal deaths, data derived from nationally coordinated facility based reviews of MNM and maternal deaths could, irrespective of the denominator of the population from which it is derived, serve as a foundation for assessing the contributions of health institutions towards the attainment of MDG-5 at a country level. The major advantage of such effort is the use of existing infrastructure for the gathering of reliable and up to date data. Concurrent nationwide enquiries into MNM and maternal deaths using uniform definitions and methods of case-identification at referral hospitals can be used to monitor the quality of comprehensive emergency obstetric care provided by capable institutions in a country. The successful implementation of this project will provide a guideline for the commencement of the long-awaited Confidential Enquiries into Maternal Deaths in Nigeria.

In view of the present level of maternal mortality and severe morbidities recorded in Nigerian hospitals, [4,8,11-13] reduction in the absolute number of maternal deaths in these institutions would be a major step towards the achievement of MDG-5 by the country. Within this context, establishment of a data system through a network of institutions would suffice for monitoring the progress towards MDG-5 while awaiting the success of efforts targeted at overcoming the existing barriers to population-based maternal mortality survey.

As part of the recommendations derived from the '*Strategic Dialogue to Reduce Maternal and Newborn Deaths in Nigeria*' in May 2008 by Nigerian reproductive health researchers and policy makers [14], this project aims to establish a national data system on MNM and maternal deaths through a network of all tertiary institutions in Nigeria. This system aims at harmonizing and building on the existing infrastructure among and within the Nigerian health institutions. The configuration of such infrastructure will assist in limiting the biases related to definitions of MNM and maternal death that are common features of facility-based reviews. Lessons learnt from the establishment of this system can be used to set up similar structures at lower levels of health care delivery in Nigeria.

## Objectives

### General

To create a national data system on MNM and maternal mortality in Nigerian public tertiary institutions. This system will conduct periodically a review of the magnitude of MNM and maternal deaths and the health service events surrounding these complications in an attempt to define and monitor the standard of obstetric care at this level of healthcare delivery. This aim will be achieved by creating a network of all tertiary obstetric referral institutions that prospectively collect specific information on potentially fatal maternal complications. The result will provide an insight into the collective tertiary health system strengths and deficiencies that could guide the formulation and or revision of obstetric policies and practices in Nigeria.

### Primary Specific Objectives

1. To estimate periodically, the frequencies of MNM and maternal deaths (MD) among women managed in public obstetric referral centres in Nigeria using uniform definitions.

2. To determine the primary determinants of MNM and cause distribution of maternal deaths.

3. To determine the case fatality rate (CFR) for direct obstetric complications and mortality index (MI) for life-threatening maternal conditions (MNM + MD) as measures of the quality of emergency obstetric care.

4. To identify areas of substandard care contributing to maternal deaths by comparing the health service events surrounding the management of women who survive (MNM) and those who die (MD).

5. To obtain regional estimation of frequencies of MNM and maternal deaths.

6. To assess existing institutional capacities for prevention of MNM and maternal deaths.

7. To develop a network and strengthen the capacity of tertiary health facilities that would participate in future epidemiological studies of benefits to the Nigerian obstetric population.

### Secondary Specific Objective

1. To evaluate the influence of health system complexities (workforce competence, health facility infrastructure, obstetrical and basic clinical services) on the frequencies of MNM and maternal deaths occurring in the institutions.

## Methods

### General outline

• This will be a nationwide cohort study that will ascertain all women who experience MNM and those who die from pregnancy, childbirth and puerperal complications according to the WHO criteria for MNM and maternal mortality among women admitted in tertiary healthcare facilities within the six geopolitical zones in Nigeria.

• Over a period of one year, women admitted for delivery or within 42 days of delivery or termination of pregnancy will be studied at all public hospitals of tertiary institution status (University hospitals and Federal Medical Centres).

• Cases of MNM and maternal deaths during the period that the women remain on admission in the facility will be identified using pre-defined criteria amongst the cohort.

• For every woman enrolled, the health service events (care pathways) within the facility will be evaluated to identify areas of substandard care/avoidable factors through clinical audit by the local research team.

• Health service events will be compared between women who experience MNM and those who die to identify institutional factors contributing to maternal death in each facility.

• Frequencies of MNM and maternal deaths among parturients will be compared between institutions across six geo-political zones and between those with less institutional capacity versus those with appropriate institutional capacity.

• A summary estimate of the frequencies of MNM and maternal deaths will be determined at intervals. Indicators of quality of care (case fatality rate, both total and cause-specific and mortality index) will be evaluated at facility, regional and country levels.

• This data system will undergo modification based on the experience gained from the pilot phase in the initial three months and publish report quarterly through the newsletter of NNRHRT.

### Setting

The proposed system will be run in consenting public tertiary healthcare institutions in Nigeria over a one-year period. Nigeria has an estimated population of 140 million inhabitants and consists essentially of six-geopolitical zones. There are 48 public tertiary hospitals (at State and Federal levels) which serve as referral centres for other health facilities within their environs (Table 1). Women with high and low risk pregnancies (with or without complications) are delivered at these hospitals under the guidance of the midwives, interns, obstetric specialist-in training and obstetricians. For the purpose of this project, data will be collected at all the tertiary health facilities that offer obstetric services. Geographic areas within each geopolitical zone and their corresponding tertiary obstetric facilities will constitute a region.

**Table 1:** Public tertiary institutions offering obstetric services according to the six geopolitical zones in Nigeria

**ZONE**	**STATE**	**INSTITUTION**
**SOUTH WEST**	Ekiti	• Federal Medical Centre, Ido-Ekiti
	Lagos	• Federal Medical Centre, Ebute Metta
		• Lagos State University Teaching Hospital, Ikeja
		• Lagos University Teaching Hospital, Idi-Araba
	Osun	• Ladoke Akintola University Teaching Hospital, Osogbo
		• Obafemi Awolowo University Teaching Hospital Complex, Ile-Ife
	Ondo	• Federal Medical Centre, Owo
	Ogun	• Federal Medical Centre, Abeokuta
		• Olabisi Onabanjo University Teaching Hospital, Sagamu
	Oyo	• University College Hospital, Ibadan
**SOUTH EAST**	Abia	• Abia State University Teaching Hospital, Aba
		• Federal Medical Centre, Umuahia
	Anambra	• Nnamdi Azikiwe University Teaching Hospital, Nnewi
	Ebonyi	• Ebonyi State University Teaching Hospital, Abakaliki
		• Federal Medical Centre, Abakaliki
	Enugu	• University of Nigeria Teaching Hospital, Enugu
		• Enugu State University Teaching Hospital, Enugu
	Imo	• Federal Medical Centre, Owerri
		• Imo State University Teaching Hospital, Orlu
**SOUTH-SOUTH**	Akwa-Ibom	• University of Uyo Teaching Hospital, Uyo
	Bayelsa	• Federal Medical Centre, Yenegoa
	Cross-Rivers	• University of Calabar Teaching Hospital, Calabar
	Delta	• Federal Medical Centre, Asaba
		• Federal Medical Centre, Onicha-Olona
		• Federal Medical Centre, Agbor
		• Delta State University Teaching Hospital, Abraka
	Edo	• University of Benin Teaching Hospital, Benin-city
		• Irrua Specialist Teaching Hospital, Irrua
	Rivers	• University of Port Harcourt Teaching Hospital, Port-Harcourt
**NORTH -CENTRAL**	Benue	• Federal Medical Centre, Markurdi
	Abuja (Federal Capital Territory)	• National Hospital, Abuja
		• University of Abuja Teaching Hospital, Gwagwalada
	Kogi	• Federal Medical Centre, Lokoja
	Kwara	• University of Ilorin Teaching Hospital, Ilorin
	Niger	• Federal Medical Centre, Bida
	Plateau	• Jos University Teaching Hospital, Jos
	Bauchi	• Federal Medical Centre, Azare
**NORTH-EAST**	Borno	• University of Maiduguri Teaching Hospital, Maiduguri
	Gombe	• Federal Medical Centre, Gombe
	Yobe	• Federal Medical Centre, Nguru
	Kaduna	• Ahmadu Bello University Teaching Hospital, Kaduna
**NORTH WEST**	Katsina	• Federal Medical Centre, Katsina
	Kano	• Aminu Kano Teaching Hospital, Kano
	Kebbi	• Federal Medical Centre, Birnin Kebbi
	Sokoto	• Usmanu Danfodiyo University Teaching Hospital, Sokoto
	Jigawa	• Federal Medical Centre, Birnin Kudu
	Zamfara	• Federal Medical Centre, Zamfara
		• Federal Medical Centre, Gusau

### Selection of subjects

#### Inclusion criteria

All women admitted for delivery or within 42 days of delivery or termination of pregnancy in the consenting health facilities during the study period will constitute the cohort for the study. Women will be included whether or not they primarily receive antenatal care and plan to deliver at the study site.

#### Exclusion criteria

Women who are brought in dead to the hospital will be noted but not be enrolled into the study. Women with incomplete data to assess the final outcome (MNM or MD), e.g. those who discharge themselves against medical advice before the resolution of their obstetric problems, will be noted but excluded from the survey.

### Data Collection

Data will be gathered continuously for a period of one year. Trained staff will conduct prospective surveillance of medical records and complete a simple individual level data form of all enrolled women within a day of their enrolment and extract information for the period that the women remain on admission in the hospital. A dedicated and trained senior house officer/resident will be responsible for data collection on a day to day basis at each institution. A hospital coordinator will supervise the data collection, resolving, completing and clarification of medical notes before data entry.

#### Individual level data (Additional file 1)

Following daily review of cases managed at each facility, women who meet the WHO criteria for MNM and those who died will be identified by trained data collectors. Data will be recorded on a simple one-page data collection form. Incomplete data in medical records will be updated by working with attending staff before patient's discharge. These data will be entered at the facility level in a database using Microsoft Excel 2003 software and electronically forwarded monthly to the regional coordinator by the hospital coordinator. Regional coordinators will then transfer the data for collation to the central coordinating unit for integration into the national database on a monthly basis.

Individual level information to be collected is specific to these study objectives and includes the following areas.

#### 1. Maternal characteristics

Sociodemographic characteristics (including age, parity, marital status, religion, educational level completed, weight, height, ethnicity and social class), booking status, pre-existing medical problem(s), pregnancy complications (e.g. preeclampsia, eclampsia, severe anaemia, preterm prelabour rupture of membranes), onset of labour (induced or spontaneous), mode of delivery (spontaneous, vaginal birth after previous caesarean section, caesarean section, vacuum extraction, forceps delivery, destructive vaginal delivery and symphysiotomy) and fetal birthweight.

#### 2. Information on maternal outcomes

(A) MNM according to the validated criteria on MNM as proposed by WHO working group on maternal mortality and morbidity classification [7] (Table 2). For each case of MNM, data will also be collected on gestational age or time of puerperium at the time of sustaining the MNM injury, timing of MNM event with respect to admission (i.e. before or during admission), primary determinant factor of MNM (the first complication in the chain of events that led to MNM analogous to the basic causes of maternal death), criteria indicative of MNM according to WHO criteria, admission to the intensive care unit (ICU), including the reason(s) for admission to the ICU (monitoring and surveillance or intensive care) and duration of stay in the ICU, fetal outcome in those associated with labour and length of hospital stay.

**Table 2:** The WHO maternal near miss criteria: a woman presenting any of the following criteria life-threatening conditions and surviving a complication that occurred during pregnancy, childbirth or within 42 days of termination of pregnancy should be considered as a maternal near miss case [7]

**Dysfunctional system**	**Clinical criteria**	**Laboratory markers**	**Management based proxies**
**Cardiovascular**	• Shock (a)	• pH<7.1	• Use of continuous vasoactive drugs (i)
	• Cardiac arrest (b)	• Lactate>5 mEq/mL	• Cardio-pulmonary resuscitation (CPR)
**Respiratory**	• Acute cyanosis	• Oxygen saturation < 90% for ≥ 60 minutes	• Intubation and ventilation not related to anaesthesia
	• Gasping (c)	• PaO2/FiO2<200 mmHg	
	• Respiratory rate >40 or <6 bpm		
**Renal**	• Oliguria non responsive to fluids or diuretics (d)	• Creatinine ≥ 300 μmol/l or ≥ 3.5 mg/dL	• Dialysis for acute renal failure
**Haematologic/** **Coagulation**	• Failure to form clots(e)	• Acute severe thrombocytopa-enia (<50,000 platelets/ml)	• Transfusion of ≥ 5 units of blood/red cells
**Hepatic**	• Jaundice in the presence of preeclampsia (h)	• Bilirubin >100 μmol/l or >6.0 mg/dL	
**Neurologic**	• Any loss of consciousness lasting >12 h (f)		
	• Stroke (g)		
	• Uncontrollable fit/status epilepticus		
	• Total paralysis		
**Alternative severity proxy**			• Hysterectomy following infection or haemorrhage

a) Shock is a persistent severe hypotension, defined as a systolic blood pressure <90 mmHg for ≥ 60 minutes with a pulse rate at least 120 despite aggressive fluid replacement (>2 L)

b) Cardiac arrest refers to the Loss of consciousness AND absence of pulse/heart beat

c) Gasping is a terminal respiratory pattern and the breath is convulsively and audibly caught.

d) Oliguria is defined as an urinary output <30 ml/hr for 4 hours or <400 ml/24 hr

e) Clotting failure can be assessed by the bedside clotting test or absence of clotting from the IV site after 7–10 minutes

f) Loss of consciousness is a profound alteration of mental state that involves complete or near-complete lack of responsiveness to external stimuli. It is defined as a Coma Glasgow Scale <10 (moderate or severe coma).

g) Stroke is a neurological deficit of cerebrovascular cause that persists beyond 24 hours or is interrupted by death within 24 hours

h) Pre-eclampsia is defined as the presence of hypertension associated with proteinuria. Hypertension is defined as a blood pressure of at least 140 mm Hg (systolic) or at least 90 mm Hg (diastolic) on at least two occasions and at least 4–6 h apart after the 20th week of gestation in women known to be normotensive beforehand. Proteinuria is defined as excretion of 300 mg or more of protein every 24 h. If 24-h urine samples are not available, proteinuria is defined as a protein concentration of 300 mg/L or more (≥ 1 + on dipstick) in at least two random urine samples taken at least 4–6 h apart

i) For instance, continuous use of any dose of dopamine, epinephrine or norepinephrine

(B) Maternal death according to the tenth revision of International Classification of Diseases (ICD-10) by the WHO [15]. For each case of maternal death, data will be collected on gestational age or time of puerperium at the time of death, onset of complication resulting in death with respect to admission (i.e. before or during admission), underlying cause of death, admission to the intensive care unit, including the reason(s) for admission to the ICU (monitoring and surveillance or intensive care) and duration of stay in the ICU, fetal outcome in those associated with labour and duration of hospital stay at death.

#### 3. Information on health services events

The care pathway of women enrolled within the facility will also be explored. Health service events of note will include time between diagnosis of the primary determinant of MNM or maternal death and definitive treatment/intervention required to save life, the level of most senior person who treat the patient and the time until the senior person arrive after admission (or after diagnosis for in-patients), and any deviation from standard management protocol. Where present, reason(s) for deviation from management protocol will also be examined and classified as administrative, patient-orientated, and medical personnel problems. Administrative problems will include cases where lack of power supply, transport and communication, essential drugs, blood for transfusion, or competent staff resulted in deviation from standard management protocol. Patient-orientated problems will include those generated by patient or her family either by way of delay in presentation to the hospital, refusal of intervention, or inability to pay for necessary services as at when due or lack of health insurance for necessary intervention. Medical personnel problems will range from delay in initial assessment by at least a senior personnel, deficiencies in promptly making correct diagnosis, inappropriate initial management plan and poor monitoring of the critically ill-patient.

#### Health facility level data

The factors that affect maternal outcomes may be determined by the varied distribution of prevailing problems and health resources. As a result of the relationship between poverty, access to health care, sociocultural characteristics of the population, such information will be included at the aggregate level. Various facility level variables will be collected to better understand the potential influence of health system complexities on MNM and maternal deaths.

#### 1. Information on health facility (Additional file 2)

A health facility classification score adapted from that used in the WHO Global survey [16,17] will be used to summarize the features of the health facilities included in the system. This scoring system was developed based on the various characteristics that will be present on health facility form and connotes the hospital capacity in relation to 1. Basic Services, 2. General Medical Services, 3. Screening Tests, 4. Emergency Obstetric Care, 5. Intrapartum Care and 6. Human Resources. Three categories will be identified – basic, comprehensive and advanced. A point will be assigned for each item that the facility had under basic category while 2 and 3 points will be assigned for items that the facility had under comprehensive and advanced categories, respectively. The total scores will be summed up to identify those with high and low institutional capacities based on an arbitrary score of 24. This cut off score represents the maximum number of points attainable if all the resources for providing basic services are available in the facility. Data on the type (University hospital, Federal Medical Centre) and location (urban, semi-urban, rural) of the facility will also be recorded on the health facility form.

#### 2. Information on obstetric unit data

Monthly record of the total number of deliveries and live births, number of women admitted during the puerperium and distribution of cases managed at the facility irrespective of the severity or final outcome (in an analogy to the basic causes of maternal death). These will serve as the denominators for calculation of the incidence of MNM and maternal death as well as cause-specific case fatality rates.

### Definition of terms

MNM will be defined as acute obstetric complication that immediately threatens a woman's survival but do not result in her death either by chance or because of hospital care she receives during pregnancy, labour or within 6 weeks after termination of pregnancy or delivery while a MNM case is a woman with at least one MNM event according to the pre-specified criteria. For identifying MNM events, we will apply the criteria proposed for identification of MNM by the WHO working group on maternal mortality and morbidity classification [7] (Table 2). Maternal death will be defined according to the tenth revision of International Classification of Diseases (ICD-10) by the World Health Organization [15].

### Quality control procedures

#### Operating manual

A study procedure manual will be developed which will describe the study in simplified terms. This manual will stress the importance of correctness and completeness of data as well as the need for strict adherence to pre-defined methods of data collection. The manual will also contain definition of all terms used in the study (e.g. MNM), acronyms and synonyms of medical and obstetric terminologies and examples of specific questions with accompanying pre-coded answers. This is important to reduce the level of heterogeneity that may be introduced into the study by data collectors.

#### Method of the data collection and entry

The instrument for data extraction is designed for precision and easy use to optimize quality and reduce erroneous entries. The data instrument will be pre-tested over a period of one-month in selected secondary health facilities in each of the six-geopolitical zones. Investigators will then record their experience on the ease of use of data extraction tool, employment of the defined terms and data collection time. At the end of the pre-test, modification will be made to data extraction instrument as required.

#### Training for data collection

The regional coordinators for the six geopolitical zones will be trained on two occasions at coordinators' meetings. Individuals responsible for coordination of the project at the respective centres (hospital coordinator) will then have a step-down training from the regional coordinator at each geopolitical zone with the support of the central coordinating committee members. Data collectors will be trained and supervised by the hospital coordinators at the various health facilities.

#### Data quality assurance

Regional coordinators will frequently visit participating hospitals and compare a random sample of medical records with their corresponding data forms. The maternal mortality and morbidity coverage in health facility will be assessed by comparing the data forms with total number of morbidities and mortalities in each centre, as independently recorded in hospital registers. The prospective surveillance by the hospital coordinator will be based on a daily visit to the obstetric ward, intensive care unit and others relevant facilities in the collaborative centre to ensure identification of all eligible cases. Data missing from medical charts shall be searched in a variety of data sources, like the hospital discharge database and antenatal and theatre records. The staff responsible for the woman's hospital care will not be told that the woman had been identified as an eligible case for the national data system on maternal deaths and disabilities in order to avoid possible biases in conduct.

### Criteria for discontinuation

Institutions that fail to comply with continuous and accurate gathering of information and those that decide to disengage their participation will be excluded from the data system. High rate of poor data quality (≥ 20%) as identified by the methods of data quality assurance will also serve as the basis for discontinuation of institutions from the national data system.

### Data management

All the data will be centrally handled by the Data Management Unit of the Centre for Research in Reproductive Health (CRRH), Sagamu, Nigeria. Data extraction forms will be scrutinized daily by the hospital coordinator or as soon as they are submitted for updating and immediate treatment of erroneous entry. The quality of extracted data will be assessed by performing duplicated record extraction in randomly selected institutions. Identified problems through such efforts will also be addressed immediately. All problems encountered (both anticipated and unanticipated problems) will be recorded in a log book by the staff responsible for data collection. Methodological problems encountered during the implementation phase will be addressed by discussion with the regional or central coordinator.

At the facility level, all data will be verified and entered into a Microsoft Excel database by the hospital coordinator. The excel file will be electronically forwarded on a monthly basis to the regional coordinator who performs the data cleaning before subsequent transfer to the central unit. Hard copies of data entry forms will be kept by the hospital coordinator for reference if questions arise from the central coordinating unit. Hospital coordinator not responding to monthly request for cases will be reminded repeatedly by email and phone. Absence of cases in a particular month will be communicated to the hospital coordinator to control for underreporting.

All data will be coded and centrally entered into a computer database using Epi Info 2002 by a statistician at the Data Management Unit of the Centre for Research in Reproductive Health (CRRH), Sagamu, Nigeria.

### Data analysis

#### Overview

Each hospital and regional coordinator is expected to have a laptop or desktop computer system with Microsoft Office software dedicated to the project. Analysis of collected data will be performed centrally on a quarterly basis. Analysis techniques will essentially focus on obtaining descriptive data including total absolute number of maternal deaths and MNM cases, incidence of maternal death and MNM cases and CFR and mortality indices at facility, regional and country levels. Women with incomplete data to assess the final outcome (MNM or MD), e.g. those who discharge themselves against medical advice before the resolution of their obstetric problems, will be excluded from the analysis.

#### Analysis plan

The aim of the primary analysis will be to provide descriptive information on the magnitude of MNM and maternal deaths and assess whether the risk of early maternal morbidity and mortality is associated with health service events and facility complexities.

#### For aim 1 (frequencies of MNM and maternal deaths)

▪ Descriptive frequencies per collaborative centre will be calculated for MNM and maternal death. Overall estimate with 95% confidence interval will be calculated. The frequencies will be separately expressed per total number of deliveries as well as per total number of live births (maternal near miss incidence ratio) at each centre. Maternal near miss incidence ratio refers to the number of MNM cases per 1,000 live births [7].

#### For aim 2 (primary determinant of MNM and cause distribution of maternal deaths)

▪ Descriptive frequencies per collaborative center will be calculated for primary determinants of MNM and causes of maternal deaths. Overall estimates with 95% confidence interval will be calculated. Proportions of MNM cases by primary determinants will be calculated.

#### For aim 3 (case fatality rate [total and cause-specific], mortality index)

Total CFR for each collaborative centre will be expressed as the proportion of women who died among all women that experienced all degrees and types of direct obstetric complications. Cause-specific CFR will be expressed as the proportion of women who died among all women that experienced a particular direct obstetric complication. In order to appreciate the standard of care provided for each determinant of MNM and maternal death, we will calculate the mortality index for each condition. This will be expressed as the number of maternal deaths resulting from a particular determinant divided by the sum of the MNM and maternal deaths occurring from such condition, expressed as a percentage [7,10]. This will reflect the proportion of each life-threatening obstetric complication, which ends in maternal death. The overall mortality index will also be determined for each institution. This refers to the number of maternal deaths divided by the number of women with life threatening conditions, expressed as a percentage [MI = MD/(MNM +MD)]. The higher the index, the more women with life-threatening conditions die (low quality of care) and vice versa.

#### For aim 4 (health service events surrounding MNM and maternal deaths)

▪ Descriptive frequency of various classes of avoidable factors in cases of MNM and maternal deaths will be calculated per collaborative center. Overall estimate with 95% confidence interval will be calculated. The proportion of the various classes of avoidable factors will be compared for cases of MNM and maternal deaths while controlling for maternal characteristics of enrolled women.

#### For aim 5 (regional estimation of MNM and maternal deaths)

▪ Descriptive frequencies for all collaborative centres within each geopolitical zone will be calculated for MNM and maternal death. Overall regional estimate with 95% confidence interval will be calculated.

#### For secondary aim 1 (influence of health system complexities on MNM and maternal deaths)

▪ Descriptive frequencies of MNM and maternal deaths stratified by institutional capacity derived from health facility classification scores. Comparison between the proportion of MNM cases and maternal deaths among institutions with low versus those of high capacity will be made to detect any difference between the two categories.

### Number of subjects and statistical power

All women managed at the tertiary obstetric units within the period of data collection in Nigeria will constitute the cohort. Women who experience MNM or die during the course of their management are the study population. Sample size will therefore not be determined a priori.

### Duration of the project

The project will run for a period of one year and continuation beyond this period is subject to availability of funds and outcome of the first phase.

Time line:

1. Development and revision of protocol: September to April 2009

2. Proposal submission to ethical review committees/organization of sites and teams: May to June 2009

3. Regional coordinators' meeting: August 2009

4. Pre-testing of data extraction instrument: August/September 2009

5. Regional coordinators' meeting to conclude training plans and system of data management:

September 2009

6. Training of data collectors: September–October 2009

7. Data Collection: November 2009 to October 2010

8. Mid-term project review: April 2010

9. Report writing: November to December 2010

### Project management

The central coordination of the project will be by the research team at CRRH, Sagamu. At each health facility, there will be a hospital coordinator and a dedicated clinician who will see to the day-to-day data collection from the health facility. There will be at least two meetings and site visits for efficient coordination of the project by regional coordinators or central coordinating staff.

Training of regional coordinators will take place at CRRH, Sagamu, Nigeria and subsequent training of hospital coordinator will take place at convenient sites within each of the six geopolitical zones after the regional coordinators' meeting. Pilot project will commence immediately after step-down trainings of hospital coordinators and data collectors by regional coordinators.

### Anticipated problems

There are three main areas of potential limitations. These include logistics for the implementation of data collection, applicability of study findings and appropriateness of outcomes.

#### Logistic problems

The execution of the project described above in Nigeria represents an enormous but achievable task. This will mostly be apparent in the efforts needed to consistently maintain the system to be developed during the period of the project and thereafter. However, the NNRHRT, with CRRH as a coordinating unit is well qualified to execute this type of project. Success will largely depend on active and continuous participation of hospital coordinators and local research team for completeness of data. Continuous survival of the system will depend on adoption and funding of the system by the Federal Government of Nigeria through the Federal Ministry of Health (FMOH). We envisage some difficulties in working with a large number of health institutions, staff, medical protocols, and records formats and fairly new definitions of terms, which could, regardless of the operation manual, produce some misclassification of MNM cases. To minimize these, we have restricted outcomes to MNM and maternal mortality and will commence data extraction as soon as the woman meets the inclusion criteria with the opportunity to review unclear or incomplete records directly with the attending medical staff.

Another important challenge will be in the selection of hospital coordinators, data collectors and data clerks for electronic data transfer. This may be quite demanding in hospitals where obstetric specialists and qualified individuals are few and situations where an obstetrician plays multiple roles from data collector to hospital coordinator may arise. This challenge will, however, be eased with the help of central coordinating committee and previous research collaborators with the CRRH in some of the centres. Other logistic problem may arise in the area of training, monitoring of data entry forms and other capacity strengthening related expenses, e.g. manual of operation, trainers, venue and transportation at regional and national levels.

#### Applicability of study findings

The project is focused on identifying life threatening maternal complications among women managed in the tertiary hospitals, and consequently we will not measure information on women who do not have hospital births at this level. Since many women are also managed at other levels of healthcare delivery, it is possible that our findings may not be applicable to other levels of healthcare delivery in Nigeria.

#### Appropriateness of outcomes

In order to reduce the burden of data collection, we have chosen to measure only short-term in-hospital maternal outcomes given the challenges in data collection involved in conducting a nationwide multicentre study. Therefore, severe medium and long-term maternal outcomes will therefore not be measured especially among women who have vaginal delivery as they tend to be discharged earlier. Considering the fact that most maternal complications occur during hospital stay, we considered it efficient to limit outcome measure to those that occur in the hospital as large number of women can be followed with relative ease and expense through assessment of their medical records. Post-discharge follow-ups are likely to be expensive, incomplete and unrealistic as postpartum visits are generally infrequent and women discharged from hospitals in Nigeria hardly re-present even when complications arise after hospital discharge.

### Other limitations

Identification of avoidable factors by personnel who are possibly part of the management team of MNM cases may likely introduce detection bias into the study.

### Expected outcomes of the study

The outcome of the study would provide useful information to the Nigerian health practitioners and policy-makers on the strengths and weaknesses of the infrastructures provided for comprehensive emergency obstetric care in Nigeria, as reflected by the magnitude of MNM and maternal deaths and identified areas of substandard care contributing to these outcomes. In view of the currently insurmountable task of obtaining population-based maternal health indices, these will serve as the starting points for reliably assessing the country's efforts towards the attainment of MDG-5. The results will be disseminated through advocacy to policy makers (FMOH, MDG Unit at the Presidency e.t.c), scientific publications, organized conferences of obstetricians and midwives and seminars and workshops within the country.

Interventions to address issues generated from the project will be promoted through advocacy with the authorities of the institutions as well their State governments through their Ministries of Health. Lessons learnt from the establishment of this data system can be used to set up similar structures at lower levels of healthcare delivery in Nigeria.

## Competing interests

The authors declare that they have no competing interests.

## Authors' contributions

OOT conceived and designed the study. OOT and AOO prepared the first draft of the protocol. Other authors critically revised and made substantive intellectual contributions to the development of the protocol.

## Supplementary Material

Additional file 1 **Appendix 3**. National Data System on MNM & Maternal Deaths – Individual Level Data Entry Form. Click here for file

Additional file 2 **Appendix 4**. Health Facility Classification Scores for the National Data System on Near Miss and Maternal Death [17]. Click here for file
