# Supplementary material for: National data system on near miss and maternal death: shifting from maternal risk to public health impact in Nigeria
Source: Reprod Health. 2009 Jun 9;6:8. doi: 10.1186/1742-4755-6-8 (PMC2702364; doi:10.1186/1742-4755-6-8)
Supplement: Additional file 1 — Appendix 3. National Data System on MNM & Maternal Deaths – Individual Level Data Entry Form. [file 1742-4755-6-8-S1.doc]

| **National Data System on MNM & Maternal Deaths**  **Individual Level Data Entry Form** |
| --- |

# IDENTIFICATION

1. Patient’s Hosp No _________ 32. Length of hospital stay:…………………

2. Date of admission _______________________ **HEALTH SERVICE EVENTS**

3. Mode of admission: (1) emergency (2) regular 33. Time between diagnosis of the primary determinant

4. Time of admission: 1) 8a.m. to 6 p.m (2) 6.01pm to 7.59 a.m. of SAMM or MD and definitive treatment……………… (mins)

5. Referral status: 1) Not referred (2) Referred before labour 34. Level of most senior person who treat the patient: (1) Intern

3) Referred during labour 4) Referred postpartum (2) midwife (3) Junior Registrar (4) Senior Registrar (5) Consultant

6. Registration status: (1) Booked (2) Unbooked 35. Time between diagnosis and attention by senior personnel……(mins)

36. Any deviation from standard management protocol: (1) No (2) Yes

**MATERNAL CHARACTERISTICS** 37. Reason for deviation from management protocol (tick as appropriate)

7. Age (years) ____________ (2) Not known ***Administrative problems***:

8. Place of Residence (1) Within 5 km (2) More than 5 km - Lack of power supply

9. Educational level completed: (1) None (2) Pry Sch. (3) Sec Sch. - Lack/inefficient transport and communication

(4) Post-Sec - Lack of life saving drugs in hospital pharmacy

10. Occupation: (1) unemployed (2) unskilled (3) semiskilled - Non-availability of required blood/blood products for transfusion

(4) professional (5) not known - Lack of competent staff required for necessary intervention(s)

11. Religion: (1) Christianity (2) Islam (3) Traditional (4) Others - Others (specify)………………………………………..

12. Marital Status (1) married (2) not married ***Patient-orientated problems***:

13. Social class: (1) low (2) middle (3) high - Delay in presentation to the hospital

14. No of living children ____________ - Refusal of medical help, advice or treatment

15. No of previous stillbirths (>28 weeks)………….. - Language barrier

16. No of spontaneous abortion (<28 weeks) _________ - Inability to pay (or lack of health insurance) for necessary intervention

- Others (specify)………………………………………..

17. No of induced abortions ___________ ***Medical personnel problems***

18. No of previous pregnancies (total) _________ - Delay in making correct diagnosis

19. No of previous C/S __________ - Delay in definitive treatment after diagnosis

20. Outcome of last preg 1) None 2) Live birth, still alive - No assessment by senior doctor

3) Live birth, deceased 4) Spontaneous abortion - Poor monitoring of patient resulting in SAMM or maternal death

- Others (specify)………………………………………..

(5) induced abortion

21. No of months between the end of the last pregnancy

(delivery or termination) and the current delivery ______

22. Pre-existing medical problems…………………………….

……………………………………………………………..

……………………………………………………………...

23. Antenatal care status: (1): None (2): Some form of ANC

(3): ANC at study site

24. No of antenatal visits at study site___________

25. Trimester of preg at booking at study site (1) first

(2) second (3) third

26. Patient’s height (cm) __________ 2) not measured

27. Weight at admission (kg) _________(2) not-measured

28. Latest PCV (or Hb) (%) __________

**MATERNAL OUTCOME**

29. Project Selection Criterion: (1) SAMM (2) Maternal death

30. Please complete attached WHO Maternal Near Miss Surveillance Form

31. Primary determinant factor(s) for SAMM or maternal death (tick as appropriate)

- Hypertensive disorders (PE/Eclampsia/HELLP syndrome)
- Haemorrhage
- Sepsis
- Dystocia
- Anaemia
- Non-obstetric complications
