# Supplementary material for: National data system on near miss and maternal death: shifting from maternal risk to public health impact in Nigeria
Source: Reprod Health. 2009 Jun 9;6:8. doi: 10.1186/1742-4755-6-8 (PMC2702364; doi:10.1186/1742-4755-6-8)
Supplement: Additional file 2 — Appendix 4. Health Facility Classification Scores for the National Data System on Near Miss and Maternal Death [17]. [file 1742-4755-6-8-S2.doc]

**Appendix IV: Health Facility Classification Scores for the National Data System on Near Miss and Maternal Death [17]**

| **Category** | **Essential (1 point each)** | **Comprehensive (2 points each)** | **Advance (3 points each)** |
| --- | --- | --- | --- |
| Basic services | Reliable water supply (1)  Toilet facilities (1)  Electricity (1)  Refrigerator (1)  Telephone (1) | Power generator (2)  Incinerator (2)  Functioning Ambulance (2) | Internet access (3) |
| General Medical Services | Biochemical/clinical laboratories (1)  Sterilization equipments (1) | Safe blood (2)  Newborn care unit with incubators (2)  Radiology department (2)  General anaesthesia equipment (2) | Adult intensive care unit (ICU) (3)  Neonatal intensive care unit (NICU) (3)  Ultrasound services (3)  Independent high risk consultation clinic (3)  Medical clinics for referral in the  same hospital (3) |
| Screening tests | Rhesus antibodies / ABO (1)  Proteinuria (1)  HIV (1)  Genotype (1)  Syphilis (1)  Urine culture (1) | Glucose tolerance test (2) | Hepatitis B (3)  Alpha fetoprotein (3)  Rubella (3) |
| Emergency  obstetric care | Availability of:  Parenteral antibiotics (1)  Parenteral oxytocic drugs (1)  Parenteral anticonvulsants for pre-eclampsia and eclampsia (1)  Neonatal resuscitation (1) | Blood transfusion (2) | Capacity for special procedures: Hysterectomy (3)  Invasive haemodynamic monitoring (3)  Mechanical ventilation (3) |
| Intrapartum  care | Partograph  Staff skilled in:  Forceps extraction (1)  Vacuum extraction (1)  Breech vaginal delivery (1)  For newborn care at delivery (1) | Staff skilled in: CS (2) | Fetal/obstetric ultrasound (3)  Electronic fetal monitoring (3) |
| Human Resources | Clinical officers (1)  Nurse anaesthetist (1)  Nurses (1)  Midwives (1) | Anaesthesiologist on call (2)  General practitioner (2) | OB/GYN specialist (3)  Anaesthesiologist available 24 h (3) |
